# Analysis of lncRNA in the skeletal muscle of rabbits at different developmental stages

**DOI:** 10.3389/fvets.2022.948929

**Published:** 2022-09-21

**Authors:** Cuiyun Y. Zhu, Qi Zheng, Qianqian Q. Pan, Jing Jing, Shuaiqi Q. Qin, Mengyu Y. Lou, Yuhang H. Yang, Jinbo B. Wei, Shuang Li, Fugui G. Fang, Yong Liu, Yinghui H. Ling

**Affiliations:** ^1^College of Animal Science and Technology, Anhui Agricultural University, Hefei, China; ^2^Anhui Province Key Laboratory of Local Livestock and Poultry Genetic Resource Conservation and Bio-Breeding, Anhui Agricultural University, Hefei, China; ^3^Key Laboratory of Embryo Development and Reproductive Regulation of Anhui Province, Fuyang Normal University, Fuyang, China

**Keywords:** lncRNA, skeletal, muscle, transcriptome, rabbits

## Abstract

It is universally acknowledged that lncRNA plays an important role in the regulation of animal skeletal muscle development regulation. However, there is a lack of relevant research on lncRNA in rabbit skeletal muscle development. Thus, we explored the expression profiles of lncRNA in rabbits at three growth stages (2-week-old fetus, 6-week-old post-weaning, and 6-month-old adult) using RNA-seq. A total of 554 differentially expressed lncRNAs (235 up- and 319 down-regulated) were found between the post-weaning and fetus groups and 19 (7 up- and 12 down-regulated) between the post-weaning and adult groups and 429 (115 up- and 314 down-regulated) between the fetus and adult. The enrichment pathways in the post-weaning and fetus groups were mainly concentrated at AMPK and PI3K-Akt signaling pathways, and the co-expression results revealed that LINC-2903, LINC-2374, LINC-8591 plays a role in early maintenance of skeletal muscle development. The enriched pathways in the fetus and adult groups were mainly involved in PI3K-Akt signaling pathways with a strong association found in mTOR signaling pathways. Analysis of the co-expression results suggests that LINC-5617 may be involved in the proliferation of embryonic skeletal muscle cells, and that LINC-8613 and LINC-8705 may provide energy for postnatal skeletal muscle development. The specific roles of different lncRNAs in different developmental stages of New Zealand White rabbits obtained. This will contribute to the subsequent study on the regulatory mechanism of muscle development in New Zealand White rabbits.

## Introduction

The New Zealand White rabbit (*Oryctolagus cuniculus*), which is native to the United States and was introduced to China many years ago, has beneficial characteristics for commercial purposes including fast early growth, and a high level of fatty meat (1). The New Zealand white rabbit has also been widely used in the biological and medical fields in recent years as an ideal laboratory animal. New Zealand white rabbit meat has been preferred by the consumers, due to its high protein levels, low cholesterol and low fat advantages (2). The leg muscle is the most economically valuable part of meat-producing rabbits and has therefore been the focus of molecular breeding efforts to improve yields from commercial rabbit farming. With the development and application of molecular biology and bioinformatics, it has become possible to understand the basis of meat rabbit production performance at the molecular level (3).

Long non-coding RNAs (lncRNAs) are >200 nucleotides non-coding RNA molecules that have been widely recognized to play important roles in many cellular processes, including the cell cycle (4), cell differentiation, metabolism, and diseases (5). For example, some lncRNAs act as molecular scaffolds by assembling protein complexes that can activate or repress transcription (6). Another portion of the lncRNAs may act as decoys to isolate transcriptional regulators and repress their activity (7). The search for lncRNAs associated with muscle developmental traits has become particularly important in commercial animal farming. Emerging research also suggests that lncRNAs play an important role in muscle development in pigs (8), calves (9), chickens (10), sheep (11), rabbits (3), and humans (12). Fifty-five differentially expressed lncRNAs were identified by RNA-seq in high and low intramuscular adipose tissues in the pig, suggesting that these molecules may be involved in muscle fat metabolism (3). LncRNAs located at meat quality trait-related loci were identified in nine muscle samples from Limousin bull calves, suggesting that they may be associated with superior meat quality (9). Differentially expressed lncRNAs have also been identified in multiple tissues in chicken skeletal muscle and specific overexpression of the lncRNA *Gallus gallus*(gga)-lnc-0181 was found to play an important role in chicken muscle development (10). LNC-011371, LNC-007561, and LNC-001728 were found to play an important role in regulating muscle development in goats during growth to adulthood (13).

We hypothesized that lncRNAs play a significant role in muscle development at different stages in rabbits but there is a lack of relevant studies. Therefore, we performed RNA-seq analysis on New Zealand white rabbits at three developmental stages (2-week-old fetus, 6-week-old post-weaning, and 6-month-old adult rabbit), explored the expression profiles of lncRNAs at the three stages, screened differentially expressed lncRNAs and obtained potential lncRNAs associated with muscle based on their functional analysis and corresponding target genes. Our current findings provide a theoretical basis for the molecular mechanisms of muscle development in meat rabbits and contribute to the development of this aspect of research.

## Materials and methods

### Experimental animal preparation and sample collection

New Zealand white rabbits in the study were purchased from the animal room of Anhui Medical University (Hefei, China), including post-weaning stage rabbits at 6-week-old (0.86 ± 0.083 kg) and adult female rabbits with 2 weeks gestation at 6-month-old (4.37 ± 0.033 kg). When sampling, muscle anesthesia was performed with Jingsongling (2,4xylyl xylazole, Lot Number 030725, Shandong Zibo Veterinary Medicine Factory, Shandong, China) at 2 mg/kg before cesarean section. The fully anesthetized rabbits were euthanized by injecting air into the ear vein. The fetuses were taken out from the uterus of female rabbits by cesarean section. Only 1 fetus (each animal represents 1 repetition) was selected for each female rabbit, that was, there were 3 biological repeats in each stage (9.14 ± 0.33 g). The left hind leg triceps brachii were taken as samples. All samples were rinsed three times with DPBS (Servicebio, Wuhan, China) containing 1 × penicillin and streptomycin (14, 15), post-weaning immediately snap frozen in liquid nitrogen (Sichuan Dongya Industry and Trade Co Ltd, Sichuan, China), and then quickly transferred to a −80°C freezer and stored until required for RNA extraction.

### Extraction and quality testing of total RNA

Extract 0.5 ± 0.1 g of rabbit tissue samples with TRIzol total RNA. (Invitrogen, Carlsbad, CA, United States). These RNA preparations were then quality tested using 1.5% agarose gel electrophoresis. And the concentration of the extracted RNAs was tested with the Nanodrop-2000 nucleic acid protein analyzer NEW (Agilent Technologies, CA, United States), and the results indicated an OD260/280 of between 1.8 and 2.1. Finally, the integrity of the RNA was measured with an Agilent 2100 bioassay instrument.

### Construction of the transcriptome cDNA library

Aliquots of 5 μg of RNA from each sample tissue were used as the starting material for library construction. Briefly, ribosomal RNA was first removed from the total RNA using an rRNA-free kit (Epicenter, Madison, WI, United States) and linear RNA was then removed with RNase R (Epicenter, Madison, WI, United States). An RNA-seq library was then generated using an NEBNext^®^ Ultra™ Directional RNA Library Prep Kit for Illumina^®^ (NEB, Ipswich, MA, United States). The library fragments were purified into cDNA fragments, with a preferred length of 250–300 bp, using the AMPure XP system (Beckman Coulter, Beverly, United States). AMPure XP beads were then used to purify the double-stranded cDNA, repair the ends of this purified cDNA, and then add poly A linkers. Finally, the cDNA library was constructed by PCR amplification.

### Clustering and quality control analysis of the RNA libraries

The index-encoded samples were clustered using the cBot cluster generation system through TruSeq PE Cluster Kit v3-cBot-HS (NEB, Ipswich, MA, United States). Illumina PE150 (pair end 150) sequencing was then performed in accordance with the effective concentration. Briefly, four fluorescently labeled dNTPs, DNA polymerase and adaptor primers were added to the sequencing flow cell for amplification. The Illumina platform then captured the fluorescent signal and converted the light signal into a sequencing peak to obtain sequence information.

Clean reads were next filtered from the original reads which were processed by the internal Perl script (ng-qc, parameter: -L 20, -p 0.5). This filter condition removes reads containing a 5′ linker and poly A/T/G/C, no 3′ linker or insert sequence, and those with an N (N means base information cannot be determined) ratio >10%. In addition, when the number of low-quality bases contained in a single-end read exceeded 50% of the length of the read, the paired reads were also removed. The Q20, Q30, and GC content of the clean data were calculated at the same time.

### Mining and analysis of the sequence data

The FA and GTF files of the reference genome and gene model of the New Zealand white rabbit (*Oryctolagus cuniculus*) were downloaded from the NCBI database (*OryCun2.0 GCA_000003625.1*). Bowtie2 v2.2.8 was used to construct the index of the reference genome and Hisat2 was employed to align the clean reads of the paired ends. LncRNAs were subsequently detected and identified using StringTie (16). The raw counts of the obtained lncRNAs were normalized using a Fragments Per Kilobase of transcript sequence per Million base pairs sequenced (FPKM) approach, which represents the expression level of the lncRNA (17). Differentially expressed lncRNAs were identified from the expression level analysis. The negative binomial distribution of DESeq2 (R-3.1.2) was used for differential analysis of the transcripts (18). The |log_2_(Fold Change)|≥1 and an adjusted *P-*value (*P-*adjust) < 0.05 were used as the threshold to screen for differentially expressed lncRNAs (19). LncRNAs with a *P*-adjust < 0.05 were designated as differentially expressed.

### Functional and pathway analysis of differentially expressed lncRNAs

Gene Ontology (GO) was used for GO enrichment analysis and Kyoto Encyclopedia of Genes and Genomes (KEGG) pathway analysis. All the obtained New Zealand white rabbits differentially expressed lncRNAs were annotated into the GO (http://www.geneontology.org) and KEGG (http://www.kegg.jp) databases. *P*-values < 0.05 indicated significant enrichment.

### Target gene prediction for the differentially expressed lncRNAs

Target gene prediction was performed on three groups (fetus, post-weaning, and adult) of differentially expressed lncRNAs. LncRNAs' target genes were predicted by the positional relationship (co-location) and expression correlation (co-expression) of lncRNAs with protein-coding genes. Then, functional enrichment analysis (GO/KEGG) was performed on the target genes of the differential lncRNAs to predict the lncRNAs associated with muscle development. A Pearson correlation coefficient (PCC) was calculated to evaluate the co-expression relationship between differentially expressed lncRNAs and mRNAs. Co-expression pairs with a PCC > 0.8 and a *P*-value < 0.05 were selected for constructing regulatory networks and were visualized using Cytoscape 3.5.1 (http://www.cytoscape.org).

### Real-time fluorescence quantitative PCR validation

qPCR was performed using a 2 × Q3 SYBR qPCR Premix (TOLOBIO, Shanghai, China) and a fluorescent qPCR instrument (Thermo, Shanghai, China). Randomly selected 10 differentially expressed lncRNAs. Primer pairs were designed using NCBI Primer-BLAST and synthesized by TsingKe Biotechnology (TsingKe, Nanjing, China). The *GAPDH* internal reference gene was used as a control. PCR reactions were conducted in a final volume of 25 μL, comprising the following: 12.5 μL of 2 × Q3 SYBR qPCR Mix (High ROX), 0.5 μL of PCR forward primer (10 μmol/L), 0.5 μL of PCR reverse primer (10 μmol/L) (Table 1), 1 μL of DNA template (cDNA solution) and 10.5 μL of sterilized water. Amplification conditions were as follows: pre-denaturation at 95°C for 2 min followed by 40 cycles of 95°C for 15 s and 60°C for 34 s. Melting curve analysis was conducted at 95°C for 15 s, 60°C for 34 s, and 95°C for 15 s.

**Table 1:** Primers and sequences of the selected lncRNAs for qPCR.

**Primer name**	**Gene-id**	**Primer sequences (5^′^–3^′^)**	**Fragment size/bp**
LINC-480	XLOC-022528	F:CACCACCTCTCTCTCACCAAG R:CCCAGCTTCTCACTAAGCCAT	180
LINC-1522	XLOC-067637	F:GGCATAGACGGGATTCTGGG R:TGAACAACCATGACACAGTGGA	102
LINC-3981	XLOC-167500	F:GTGCTTCTCCTGGTCTCCCAT R:GTGCTGGATTCTGTCCTGGTT	122
LINC-8206	XLOC-309022	F:TGTCCCTTTGTGCTTGGTCA R:GCAAATGCTTGTGACGGGTG	200
LINC-8653	XLOC-318854	F:CAGCAGCCATCCGGTATCAT R:GGGAAGGGTTTCTGATGCGA	157
LINC-8705	XLOC-319783	F:TGCAAGTCTACCGTGTGAGT R:AGCTTGGTGGAGCATTGAGT	140
LINC-8943	XLOC-323492	F:AACCAATTAGCGTTTGGCAGC R:TGACTCCAGGTGGAACCAAT	117
LINC-2374	XLOC-105852	F:AAAGTTCCCAGTCCCCAAGC R:TGATCCACAGGTCGTTTGCT	143
LINC-5363	XLOC-224411	F:CCGCTTGTGAAGGGCTGTAA R:GTCTTCGGTCCTGAGCAAGT	101
SPO11-AS1	100359270	F:TCAGCTCGATGGCGCATTTA R:AAATGATCTGACGACGCCCA	107
LINC-2903	XLOC_122121	F:CATGGCTTTTTGCCACTCCA R:ACGAAGTGGCCCTGTCTTTC	154
LINC-8591	XLOC_317430	F:GGGAAATGGAGTCCCCCAAG R:GGAAATCCTGATCCAGGGGAC	97
LINC-8613	XLOC_317978	F:GTTCTAGTCCTGGTTGGGGC R:CCTTTTCCGTTGGTTCAGCC	119
LINC-5617	XLOC_236837	F:GGATGACCACTTCGACCCAG R:CGCGTAGTGATAGGCCAACT	212

Annotate: SPO11-AS1 for SPO11 initiator of meiotic double stranded breaks.

F, Forward; R, Reverse.

### Data analysis

The lncRNA expression levels were normalized to a reference sequence and calculated by 2^−ΔΔCt^. The data were all initially collated using Excel 2016, and analyzed by IBM SPSS Statistics v19.0 for one-way ANOVA, which was performed on the normalized data using GraphPad Prism 8.0, using the analysis correlation bi-variables method to analyze the relationship between muscle development-related gene expression and muscle phenotype data. A Spearman correlation coefficient was used (20). *P* < 0.05 was used as the criterion for significant differences.

## Results

### RNA quality testing

Testing of the RNA *via* 1.5% agarose nucleic acid electrophoresis revealed three clear bands, among which the 28S band was the brightest, indicating that the RNA quality met the requirements for library construction (Supplementary Figure S1).

### Analysis of lncRNA sequencing data

**Figure 1:**
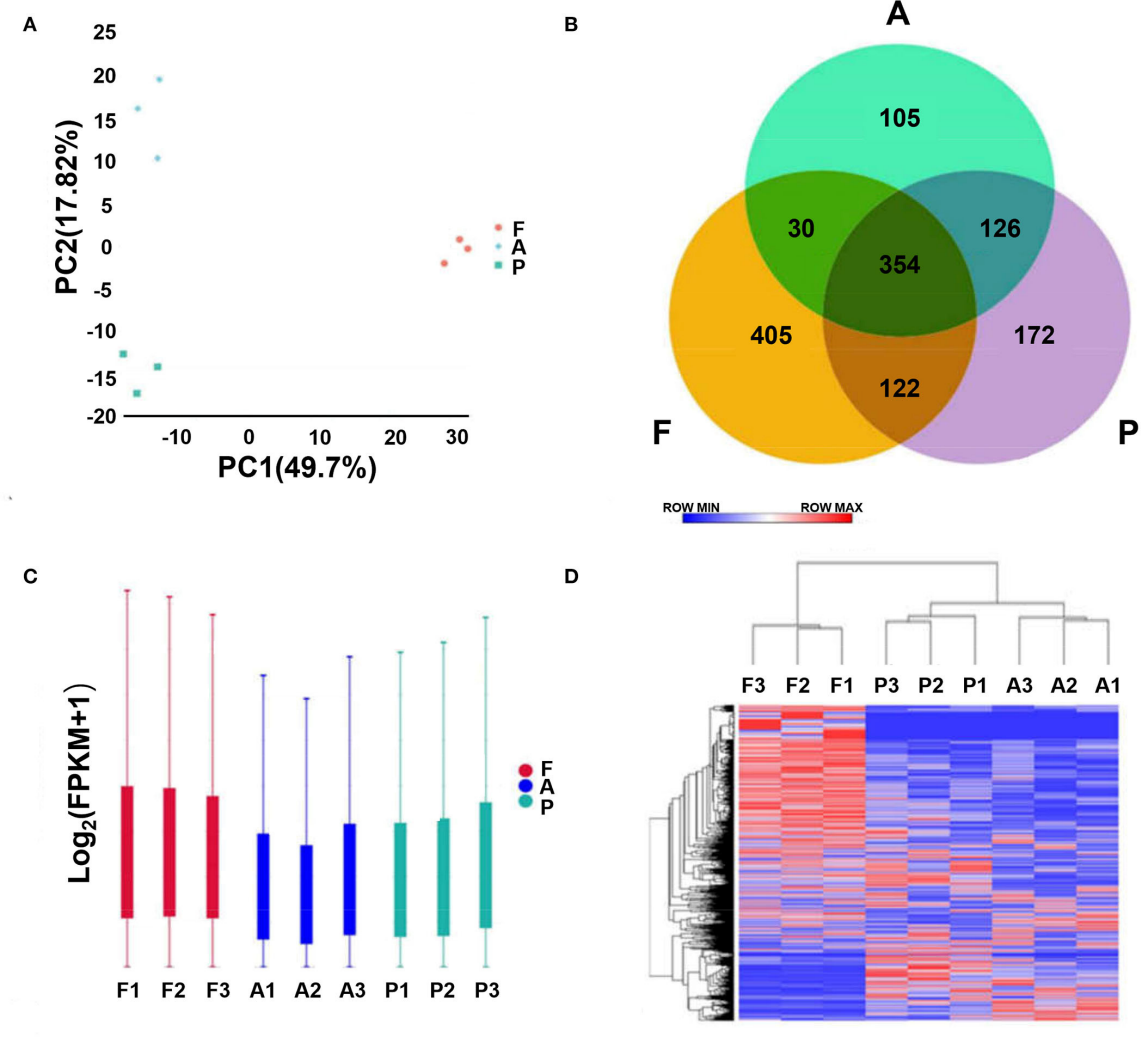
Analysis of lncRNA sequencing data. **(A)** Principal component analysis plot (horizontal coordinate is PC1, vertical coordinate is PC2, indicating the degree of clustering). **(B)** Venn diagram (where circles represent samples, non-overlapping part is specific expression lncRNA, overlapping part is non-specific expression lncRNA). **(C)** Box plot of lncRNA expression levels [horizontal coordinate is sample name, vertical coordinate is log_10_(FPKM+1), and the maximum, upper quartile, median, lower quartile and minimum values are shown]. **(D)** Hierarchical clustering plot of differential lncRNA expression in different experimental groups [each row represents one lncRNA, each column represents one sample, and the color from blue to red indicates log_10_(FPKM+1) from large to small]. (**A–D**, F, Fetus; P, Post-weaning; A, Adult).

Principal component analysis (PCA) showed clustering of the three samples from each stage, indicating reproducibility of the data. Cluster plots for the fetus, post-weaning and adult groups show reproducibility of the samples (Figure 1A). From the Venn diagram, 354 lncRNAs were revealed to be expressed in all three groups, 405 specifically in the post-weaning group, 172 specifically in the fetus group, and 105 specifically in the adult group, indicating a dramatic change in the lncRNA regulatory network in skeletal muscle between the postnatal and post-weaning stages (Figure 1B). The overall lncRNA expression levels were more homogeneous among the three stages (Figure 1C). The heat map revealed the dynamic changes in all samples (Figure 1D).

### Differential expression of lncRNAs

**Figure 2:**
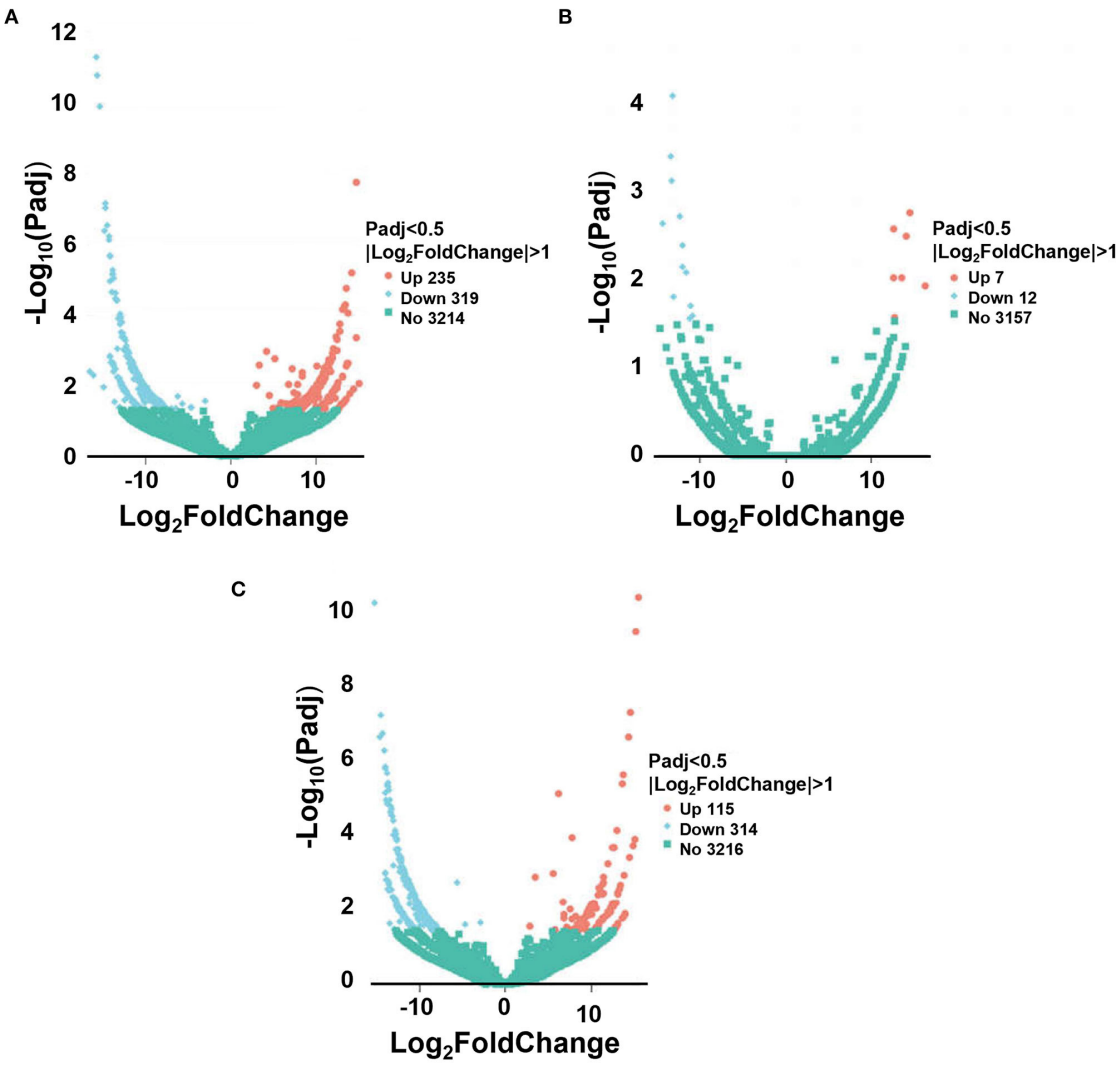
Sequencing of differentially expressed lncRNA. **(A)** Volcano plot of differential lncRNA between post-weaning and fetus groups. **(B)** Volcano plot of differential lncRNA between post-weaning and adult groups. **(C)** Volcano plot of differential lncRNA between fetus and adult groups. **A–C** Horizontal coordinates represent lncRNA expression fold changes in different samples, vertical coordinates represent statistical significance of differences in lncRNA expression changes, differentially expressed lncRNA are indicated by blue dots (up-regulated) and red dots (down-regulated) insignificant lncRNA are indicated by purple and blue dots.

To further explore the differences in the regulatory network between the 3 stages, differential analysis of lncRNA was performed. There were 554 differentially expressed lncRNAs found between the post-weaning and fetus groups (Supplementary material 4), of which 235 were up-regulated and 319 were down-regulated (Figure 2A); 19 differentially expressed lncRNAs were found in the post-weaning and adult groups (Supplementary material 5), of which 7 were up-regulated and 12 were down-regulated (Figure 2B); and 429 differentially expressed lncRNAs were found in the fetus and adult groups (Supplementary material 6), of which 115 were up-regulated and 314 were down-regulated (Figure 2C).

### GO enrichment analysis of differentially expressed lncRNAs

**Figure 3:**
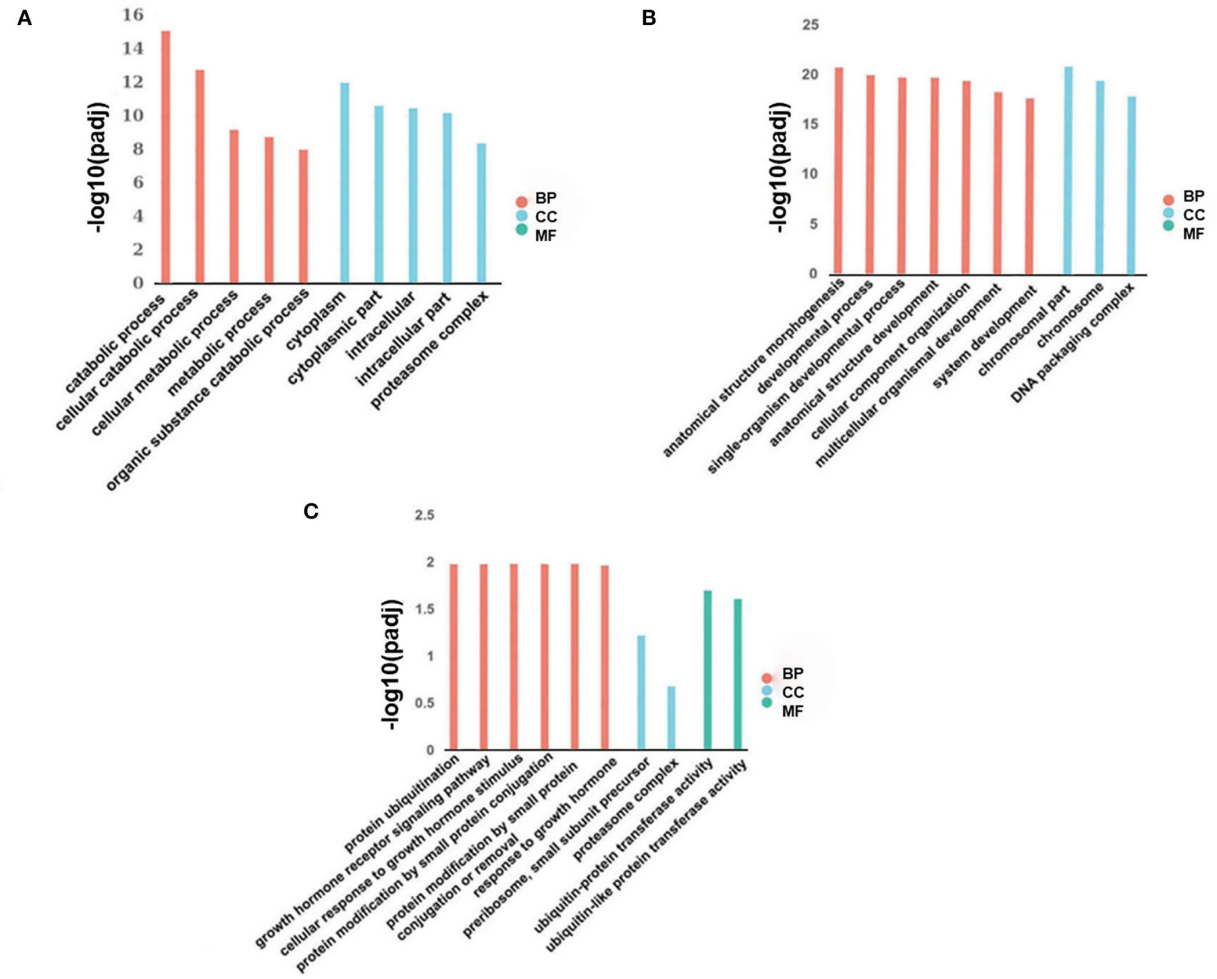
Differentially expressed lncRNA GO analysis. **(A)** Histogram of up-regulated differential lncRNA GO enrichment in post-weaning and fetus groups. **(B)** Histogram of down-regulated differential lncRNA GO enrichment in post-weaning and fetus groups. **(C)** Histogram of down-regulated differential lncRNA GO enrichment in fetus and adult groups (horizontal coordinates represent enriched GO terms, vertical coordinates represent the number of differentially expressed lncRNA in the entry). Different colors are used to distinguish biological processes, cellular components, and molecular functions; BP, biological process; CC, cellular component; and MF, molecular function.

We employed GO to functionally enrich for differential lncRNAs from two adjacent phases (fetus and post-weaning, post-weaning, and adult), and these differentially expressed lncRNAs were found to be significantly enriched in biological processes, cellular components and molecular functions. Up-regulated differential lncRNAs between the post-weaning and fetus groups were enriched in 212 GO terms (*P* < 0.05), mainly involved in precursor metabolite and energy production, ATP metabolic processes, growth hormone receptor signaling, the JAK-STAT cascade involved in growth hormone signaling pathway, and the regulation of myoblast differentiation (Figure 3A; Supplementary material 7). Down-regulated differential lncRNA enrichment was evident for 362 GO terms (*P* < 0.05), including chromosomal components, limb morphogenesis, limb development, chromosome development and skeletal formation (Figure 3B; Supplementary material 7). Up-regulated differential lncRNAs were not significantly enriched for terms between the post-weaning and adult groups. However, membrane lipid metabolism processes as well as other hormone intertransport processes were involved. Down-regulated differential lncRNA enrichment was observed in 17 terms (*P* < 0.05) including growth hormone receptor signaling, the cellular response to growth hormone stimuli, the JAK-STAT cascade involved in growth hormone signaling pathways, and in processes associated with protein modifications and growth hormone response (Figure 3C; Supplementary material 8).

### KEGG pathway analysis of differentially expressed lncRNAs

**Figure 4:**
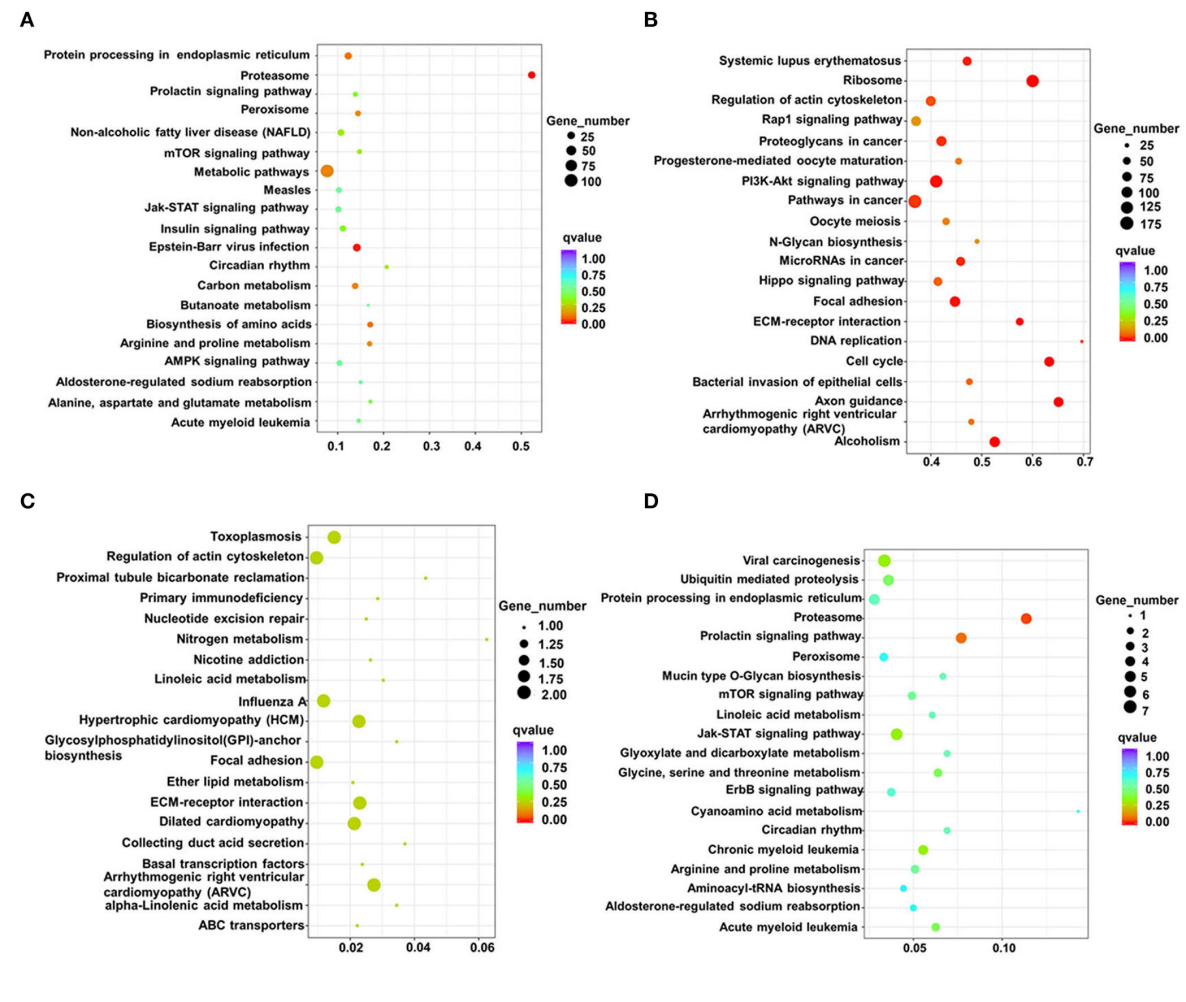
Analysis of differentially expressed lncRNAs using KEGG. **(A)** Scatterplot of all differentially up-regulated lncRNA KEGG enrichment in post-weaning and fetus groups. **(B)** Scatterplot of all differentially down-regulated lncRNA KEGG enrichment in post-weaning and fetus groups. **(C)** Scatterplot of all differentially up-regulated lncRNA KEGG enrichment in fetus and adult groups. **(D)** Scatterplot of all differentially down-regulated lncRNA KEGG in fetus and adult groups. lncRNA KEGG enrichment scatterplot. (Horizontal coordinates indicate enrichment factors, vertical coordinates indicate pathway names, the size of the dots indicates how many differentially expressed lncRNA are in this pathway, and the colors of the dots correspond to different q-value ranges of up-regulated differential lncRNA KEGG enrichment scatter plots).

The identified signaling pathways were subsequently analyzed using KEGG for differential lncRNAs in two adjacent stages (fetus and post-weaning, post-weaning, and adult). Up-regulated differential lncRNAs between the post-weaning and fetus groups were enriched in 2 pathways (*P* < 0.05) the proteasome and Epstein-Barr virus infection. However, there was also a high involvement in muscle development-related mTOR signaling and 5′-adenosine monophosphate activated protein kinase (AMPK) signaling (Figure 4A; Supplementary material 7). Down-regulated differential lncRNA enrichment was evident in 13 pathways (*P* < 0.05), mainly associated with axon guidance, phosphatidylinositol 3-kinase/protein kinase 3 (PI3K)-Akt signaling, and actin cytoskeleton regulation (Figure 4B; Supplementary material 7). Up-regulated differential lncRNAs between the post-weaning and adult groups were not significantly enriched in any pathway, but showed an involvement in myocardial development such as arrhythmogenic right ventricular cardiomyopathy (ARVC) and ECM-receptor interaction hypertrophic cardiomyopathy (HCM) (Figure 4C; Supplementary material 8). Down-regulated differential lncRNA enrichment was observed in a single pathway (*P* < 0.05), the proteasome, but were highly involved in Jak-STAT and mTOR signaling (Figure 4D; Supplementary material 8).

### Co-expression analysis of differential lncRNAs and their target genes

**Figure 5:**
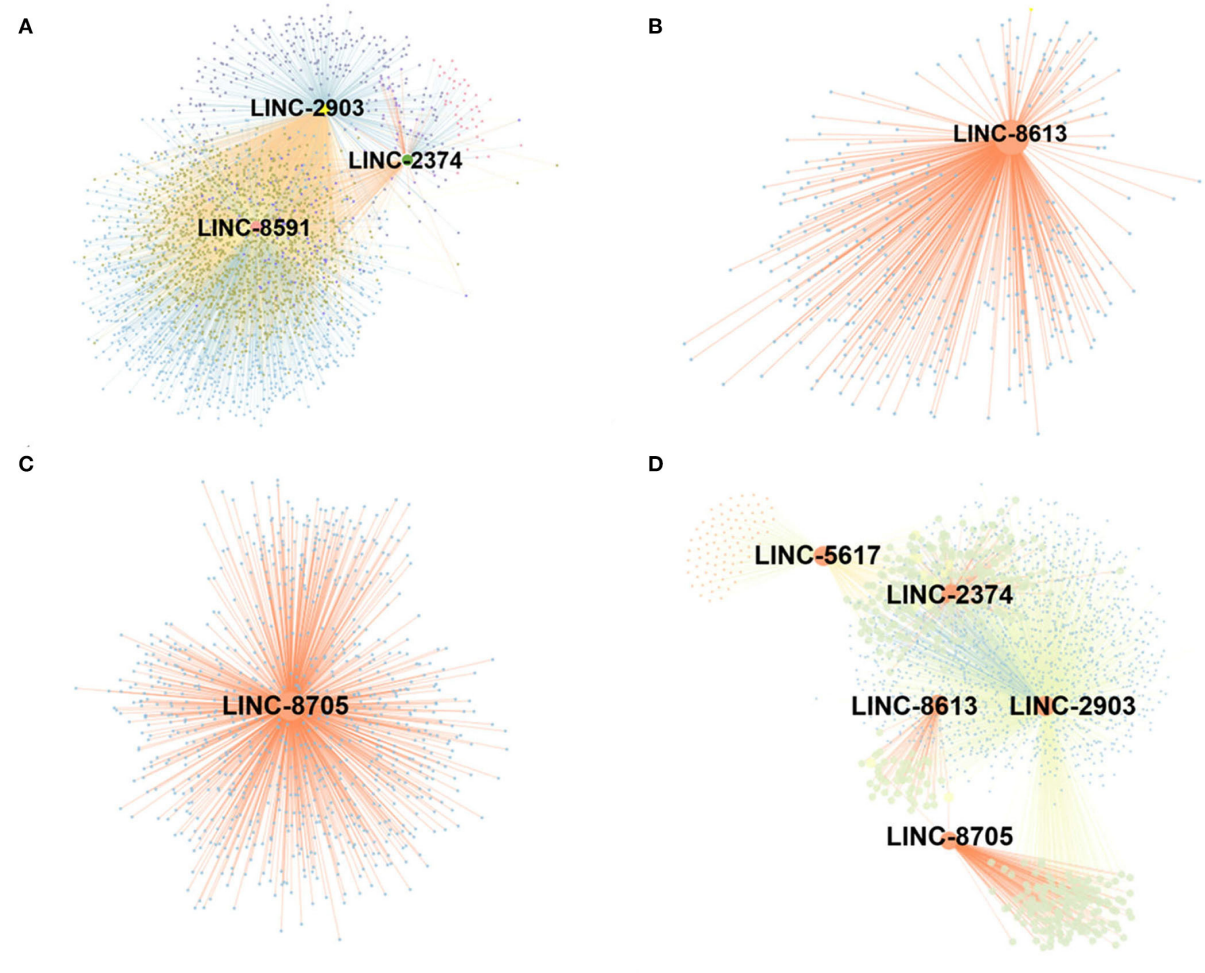
Construction of lncRNA-mRNA co-expression network. **(A)** Differential lncRNA-mRNA functional network between post-weaning and fetus groups. **(B–D)** Differential lncRNA-mRNA functional network between fetus and adult groups (large nodes represent lncRNA and small nodes represent target genes. The lines represent the regulatory relationship between lncRNA and mRNA. For the correlation between lncRNA and target genes, Pearson correlation coefficient (PCC) > 0.8, *p*-value < 0.05.

A gene co-expression network map was constructed based on the similarity of gene expression data. A total of 9 differential lncRNAs targeting 4,344 genes were identified in the post-weaning and fetus groups. Among these molecules, LINC-2903, LINC-2374, and LINC-8591 were found to be the most abundant target genes and significantly associated with muscle development. Sixteen differential lncRNAs targeting 3,665 genes were identified in the post-weaning and adult groups. among them, LINC-8613, LINC-8705, LINC-5617, LINC-2374, and LINC-2903 target genes were the most numerous and require special attention (Figure 5).

### Real-time fluorescence quantitative PCR

**Figure 6:**
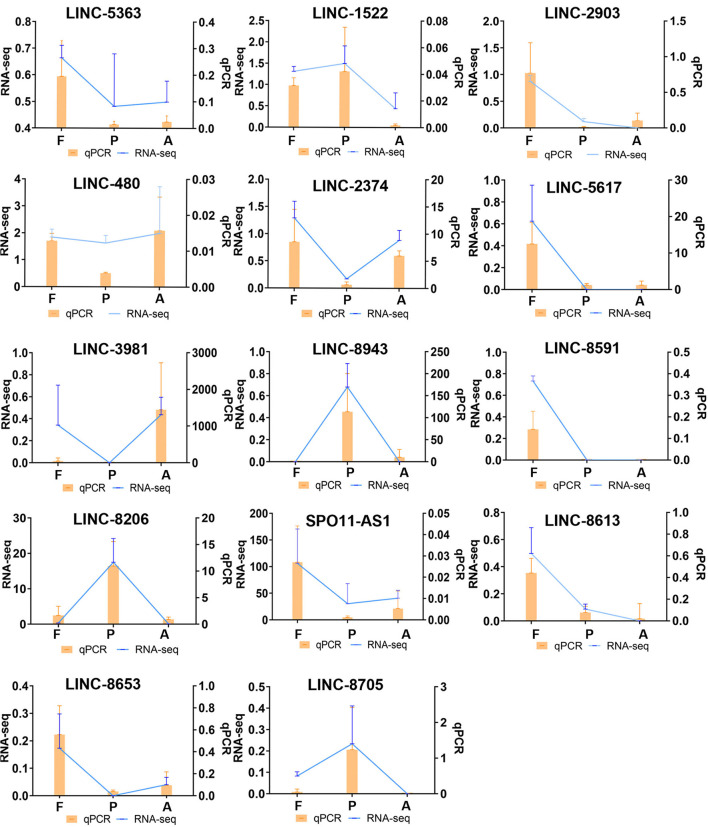
Comparison of differentially expressed lncRNA qPCR and RNA-seq results. (qPCR expression, orange, bar graph. RNA-seq expression, blue, line graph. F, Fetus; P, Post-weaning; A, Adult).

To verify the reliability of our data, high expression differential lncRNAs were screened using qPCR validation. qPCR results were given as relative expression values, and RNA-seq results as Fragments Per Kilobase of exon model per Million mapped fragments (FPKM) values. By calculating the correlation coefficient between these two data sets, the qPCR data were found to be consistent with the expression trends from the sequencing data in this study (Figure 6).

## Discussion

Postnatal skeletal muscle growth is mainly achieved through increases in the length and circumference of muscle fibers, and not by an enhanced number of muscle fibers. Hence, identifications of the key lncRNAs that function at different stages of skeletal muscle development will be important for the future development of more optimal meat production in rabbits. LncRNAs play a pleiotropic role in skeletal muscle production as key regulators of biological processes such as cell growth, development, differentiation and disease (21). However, the changes in lncRNA expression levels before and after birth in rabbits had not been clear previously. We thus conducted RNA-seq analysis of hind leg muscle samples from New Zealand white rabbits at the fetal, post-weaning, and adult growth stages to identify lncRNAs that may have potential effects on skeletal muscle development at these different stages. The data from PCA and heat map analysis showed reproducibility at each stage, and qPCR results verified the reliability of these data. Differentially expressed lncRNAs were detected between the post-weaning and fetus groups and the post-weaning and adult groups than the fetus and adult groups, with a total of 554 differentially expressed lncRNAs for the post-weaning and fetus groups, 429 for the post-weaning and adult groups, and 19 only between the fetus and adult groups. This demonstrated that the muscle development of New Zealand white rabbits is mainly concentrated in the post-weaning to fetus stages.

Our post-weaning and fetal samples were analyzed by GO and KEGG to reveal differentially up- and down-regulated lncRNAs, which were found to be predominantly enriched in myofibril formation. AMPK and PI3K-Akt signaling pathways were predominantly enriched in the post-weaning group compared to the fetus group. Prior studies have demonstrated that AMPK is an intracellular sensor of ATP consumption and a key regulator of skeletal muscle metabolism, mainly involved in promoting glucose and fat oxidation, and ATP catabolism (22). The PI3K-Akt signaling pathway is known to be involved in cell proliferation, differentiation, invasion, and apoptosis (23). Prior experiments have revealed that the activation of PI3K-Akt signaling is required for the proliferation and differentiation (24). This suggests that AMPK and PI3K-Akt signaling pathways play an important role in regulating skeletal muscle cell development in rabbits. Our current co-expression analysis in the post-weaning and fetus groups of New Zealand white rabbits identified three differentially expressed lncRNAs, LINC-2903, LINC-2374, and LINC-8591, that showed a significant association with muscle development. It has been found previously that insulin-like growth factor (*IGF*) positively regulates muscle differentiation and can stimulate myoblast proliferation (25). Myogenic (*MYOD*) (a marker gene for satellite cell activation and differentiation) (26), and Myogenin (*MYOG*) (a marker gene for late satellite cell differentiation) promote skeletal muscle satellites towards myogenic differentiation and fusion to form multinucleated muscle fibers (27). A *MYOG* knockdown reverses terminal myoblast differentiation (28, 29). The myosin heavy chain family (*MYH19* and *MYH8*) and the troponin family (*TNNT1* and *TNNT2*), which are related to the formation of myofibril types, are involved in muscle proliferation, differentiation and structure (13). LINC-2903 is associated with *IGF, MYH10, MYH8, MYOG, MYOD*, and *TNNT1*, indicating that this lncRNA molecule plays a role in maintaining normal muscle development and promoting proliferation or differentiation in the early stages of skeletal muscle development. It has been shown previously in a human study that when the plasma protein 2 (*PAPPA2*) gene is mutated or missing in children, it usually results in a short stature (30). In knockout mice for this gene, body length and bone length are lower than normal mice, and osteogenesis is abnormal (30, 31). LINC-2374 directly targets *PAPPA2* and was found in our current analyses to be highly expressed in the post-weaning stage, suggesting its involvement in rapid muscle development and a direct or indirect role in the regulation of skeletal muscle development. *MYH3* is a genetic isoform of myosin heavy chain (*MyHC*), which has been shown to form filaments in transverse, smooth and non-muscle cells and to play an important role in human infancy development and muscle regeneration (32). *MYH3* has been associated with LINC-8591 and its high expression in early development would indicate an involvement in muscle fiber formation during the progression to adulthood.

Differentially expressed lncRNAs were found to be up-regulated and down-regulated by GO and KEGG analysis in the post-weaning and adult groups in our current study. and were mainly enriched in muscle cell development and PI3K-Akt signaling processes, also highly involved in the mTOR pathway, which is clearly linked to muscle development. The PI3K-Akt signaling pathway and steroid biosynthesis are particularly prominent in these processes, which indicates their significant role in postnatal muscle growth (33). The protein mTOR is a serine/threonine kinase that regulates growth, development, and behavior by regulating protein synthesis, autophagy, and a variety of other cellular processes in response to changes in nutrients and other cues. mTOR complexes are widely described as signaling systems that sense levels of various nutrients, energy, and growth factors, and direct downstream activities such as development, reproduction, metabolism, behavior, stress responses, and aging (34). Co-expression analysis of fetus and adult group, identified three differentially expressed lnRNAs associated with muscle development, named LINC-5363, LINC-8613, and LINC-8705.

Myostatin (*MSTN*) was originally identified in mice, with mutations in this gene resulting in skeletal muscle hypertrophy, and is known to negatively regulate muscle development (35). LINC-5363 directly targets *MSTN*, suggesting that this lncRNA may play a negative regulatory role from fetal to adult muscle development in animals. It has been reported that *Myh10* is a pleiotropic gene that plays multiple roles in different developmental processes, including tension production (35), growth factor receptor internalization (36), cell adhesion (37) and extracellular matrix protein secretion (38). *Myh10* encodes a non-muscle myosin heavy chain *IIB* (*NMHC IIB*), which has been shown to be a cytoskeletal protein with multiple functions, includingcytoplasmic division, cell shape regulation, adhesion and migration (39). *NMHC IIB* plays a critical role in cytoskeletal formation. LINC-5617 targets *MYH10*, suggesting its possible involvement in the proliferation and development of skeletal muscle cells. Myosin heavy chain (*MyHC*) is the molecular motor of muscle and forms the backbone of the thick filaments of myofibrils (33). Different *MyHC* isoforms are important for the physiological properties of different muscle fiber types. Dominant mutations in the developmental *MyHC* isoform gene (*MYH8*) are associated with distal joint flexure syndrome. Myosin is a highly conserved and ubiquitous protein present in all eukaryotic cells (40). It acts as a molecular motor converting the chemical energy of ATP hydrolysis into mechanical forces for various cellular motilities such as cytoplasmic division, phagocytosis and muscle contraction (41). LINC-8613 is associated with *MYH8* and is primarily associated with signaling pathways that provide energy, suggesting that postnatal LIN-8613 is primarily associated with cellular energy supply. Protein kinase cAMP-dependent type II regulatory subunit beta (*PRKAR2B*) enhances glucose consumption, lactate production and the rate of extracellular acidification (42). *PRKAR2B* increases the expression level of hypoxia-inducible factor 1α (HIF-1α), suggesting that *PRKAR2B* can consume large amounts of energy, which is closely related to skeletal muscle development (43). The transcription factor *ZBTB14* affects β-catenin protein content by reducing junctional β-catenin protein mRNA levels, which in turn exerts an inhibitory effect on Wnt/β-catenin signaling, thereby exerting a regulatory effect on myofiber hypertrophy (44). LINC-8705 is associated with *PRKAR2B, ZBTB14*, and is highly expressed in the post-weaning, suggesting that it may play an important role in bone muscle development after birth in New Zealand white rabbits.

## Conclusion

We analyzed for the first time skeletal muscle tissue from the hind legs of New Zealand White rabbits at three developmental stages (2-week-old fetuses, 6-week-old post-weanings, and 6-month-old adults) using RNA-seq. Co-expression analysis of the fetal and post-weaning groups showed that LINC-2903, LINC-2374, and LINC-8591 lncRNAs played a role in maintaining normal muscle development and promoting proliferation or differentiation in the early stages of skeletal muscle development. Co-expression results in post-weaning and adult groups suggest that LINC-5617 may be involved in skeletal muscle cell proliferation and development. LINC-8613, and LINC-8705 are mainly associated with signaling pathways that provide energy, suggesting that they may provide energy for postnatal skeletal muscle development. Thus, our current study provides a solid foundation for further studies on muscle developmental characteristics and meat improvement in New Zealand white rabbits, as well as broader studies on muscle development in herbivores.

## Data availability statement

The datasets presented in this study can be found in online repositories. The names of the repository/repositories and accession number(s) can be found in the article/Supplementary material.

## Ethics statement

All experiments were performed in accordance with relevant guidelines and adhere to the ARRIVE guidelines (https://arriveguidelines.org/) for the reporting of animal experiments. This study was carried out in accordance with the principles of the Basel Declaration and Recommendations of the Guide for the Care and Use of Laboratory Animals (http://grants1.nih.gov/grants/olaw/references/phspol.htm). The protocol was approved by the Ethics Committee of Anhui Agricultural University under Permit No. AHAU20101025.

## Author contributions

YLin conceived and designed the experiments and provided acquisition of the financial support for the project leading to this publication. CZ and QZ performed the experiments and analyzed the data. QZ, CZ, and QP contributed reagents, materials, analysis tools. QZ, CZ, JJ, and SQ wrote the article. YLin, SL, YLiu, and FF revised the manuscript. All authors read and approved the final manuscript.

## Funding

This research was supported by the National Natural Science Foundation of China (32172695), the Natural Science Foundation of Anhui Province (2108085Y11), the 2021 Annual Mutton Goat Industry Development Pilot Technology Project of Linquan County (LQRJK2021-03), and Open Project of Anhui Key Laboratory of Embryonic Development and Reproductive Regulation (2022).

## Conflict of interest

The authors declare that the research was conducted in the absence of any commercial or financial relationships that could be construed as a potential conflict of interest.

## Publisher's note

All claims expressed in this article are solely those of the authors and do not necessarily represent those of their affiliated organizations, or those of the publisher, the editors and the reviewers. Any product that may be evaluated in this article, or claim that may be made by its manufacturer, is not guaranteed or endorsed by the publisher.

## Abbreviations

LncRNA: Long non-coding RNA
FPKM: fragments Per Kilobase of transcript sequence per Millions base pairs sequenced
GO: Gene Ontology
PCA: Principal component analysis.
