# Supplementary figures and images for: Analysis of lncRNA in the skeletal muscle of rabbits at different developmental stages
Source: Front Vet Sci. 2022 Sep 21;9:948929. doi: 10.3389/fvets.2022.948929 (PMC9533132; doi:10.3389/fvets.2022.948929)

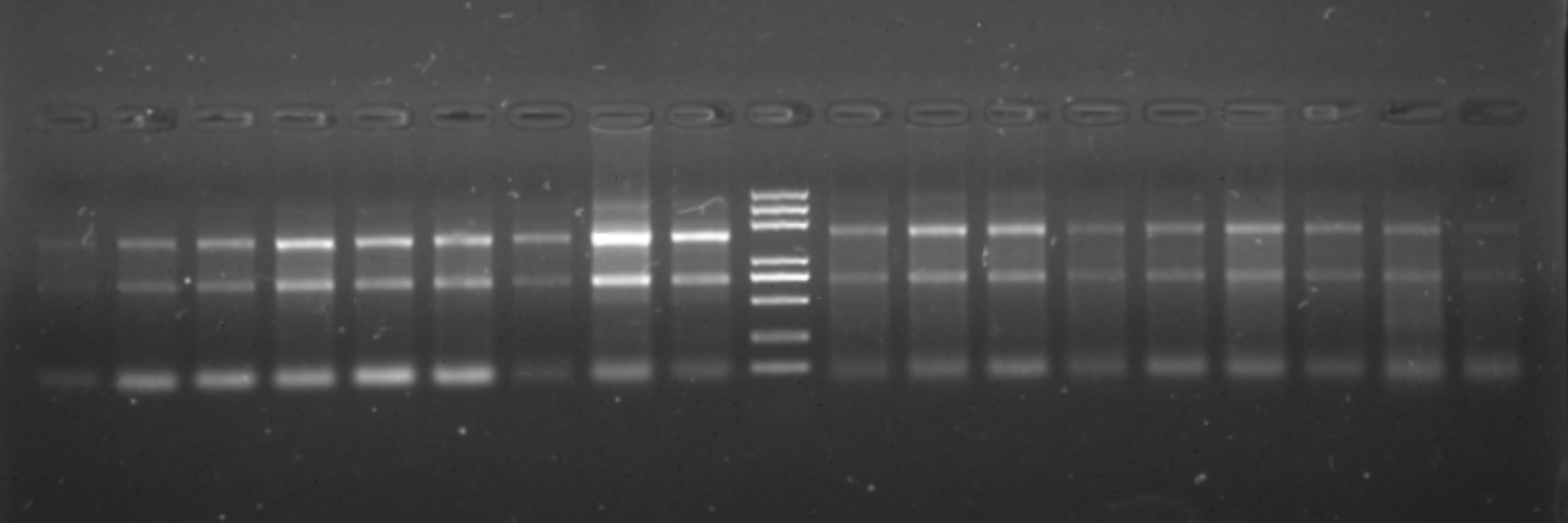

Supplement: Supplementary file 9 [file Image_1.JPEG]
